# Poor Karnofsky performance status is not a contraindication for neurosurgical resection in patients with lung cancer brain metastases: a multicenter, retrospective PSM-IPTW cohort study

**DOI:** 10.1007/s11060-023-04293-8

**Published:** 2023-03-20

**Authors:** Lun Liang, Liangbao Wen, Shixing Qin, Zhenqiang He, Jie Lu, Run Cui, Xiaobing Jiang, Hongrong Hu, Sheng Zhong, Chang Li, Chengwei Yu, Yuang Xie, Zhenning Wang, Hao Duan, Yonggao Mou

**Affiliations:** 1 Department of Neurosurgery, State Key Laboratory of Oncology in South China, Collaborative Innovation Center for Cancer Medicine, Sun Yat-sen University Cancer Center, Dongfeng Dong Road, Guangzhou, 510000 China; 2 Department of Neurosurgery, Dongguan People’s Hospital (Affiliated Dongguan Hospital, South Medical University), Dongguan, 523058 China; 3 Department of Neurosurgery, Guangxi Medical University Cancer Hospital, Nanning, 530021 China; 4 Department of Cerebrovascular Disease and Spine Neurosurgery, The People’s Hospital Of Guangxi Zhuang Autonomous Region, Nanning, 530016 China

**Keywords:** Neurosurgical resection, Lung cancer brain metastases, Karnofsky performance status

## Abstract

**Backgound:**

Neurosurgical resection is a standard local treatment for lung cancer brain metastases (BMs). This study aims to investigate whether neurosurgical resection provides survival benefit in lung cancer BMs with poor KPS.

**Materials and methods:**

This multicenter retrospective study included 386 lung cancer BMs with pretreatment KPS ≤ 70 among a total of 1177 lung cancer BMs treated at three centers from August 2010 to July 2021. Data analysis was performed from July to September 2022. Inverse probability of treatment weighting (IPTW) and propensity scores matching (PSM) based on propensity scoring were used to minimize bias. The main outcome was overall survival (OS) after diagnosis of BMs. Risk factors of OS were estimated using Cox proportional hazards regression models. All Characteristics were included in the multivariate Cox regression.

**Results:**

386 patients with pretreatment KPS ≤ 70 were included (age mean [SD], 57.85 [10.36] years; KPS mean [SD], 60.91 [10.11]). Among them, 111 patients received neurosurgical resection, while 275 patients did not. Baseline characteristics were balanced between groups after IPTW or PSM. Neurosurgical resection was associated with significantly better prognosis in unadjusted multivariate COX analysis (hazard ratio [HR]: 0.68, 95% confidence interval [CI]: 0.51–0.91, *P* = 0.01), and PSM-adjusted multivariate COX analysis (HR: 0.61, 95%CI: 0.39–0.94, *P* = 0.03), IPTW-adjusted multivariate COX analysis (HR: 0.58, 95%CI: 0.40–0.84, *P* = 0.004). OS was significantly longer in neurosurgical resection group compared with non-surgical resection group according to unadjusted data (Median OS, surgery vs non-surgery, 14.7 vs 12.5 months, *P* = 0.01), PSM-adjusted data (median OS, 17.7 vs 12.3 months, *P* < 0.01) and IPTW-adjusted data (median OS, 17.7 vs 12.5 months, *P* < 0.01).

**Conclusions:**

Neurosurgical resection was associated with improved survival in patients with lung cancer BMs with poor KPS, suggesting that poor KPS is not a contraindication for neurosurgical resection in these patients.

**Supplementary Information:**

The online version contains supplementary material available at 10.1007/s11060-023-04293-8.

## Introduction

Lung cancer accounts for up to 56% of all brain metastases (BMs), with lung cancer being the most common primary cancer in BMs. Lung cancer brain metastasis is associated with high morbidity and limited survival [1], and brain dissemination is the most common cause of tumor-related death in patients with BMs [2]. The main treatment modalities, including surgical resection, stereotactic radiosurgery (SRS), whole-brain radiotherapy (WBRT), and systemic therapy, have been reported to improve survival and local control of BMs [3–6].

KPS has been widely used to assess the activity, work, and self-care abilities of cancer patients for decades [7]. Several studies have demonstrated that a poor KPS predicted poor outcomes in patients with BMs, including lung cancer BMs [8–10]. Most clinical randomized and non-randomized controlled trials have thus used a poor KPS as an exclusion criterion to reduce confounding bias [11], and treatments for BMs in patients with poor KPS have not been well-studied.

Surgical resection has become a standard local treatment for BMs and has demonstrated survival benefits in patients with large and single BMs [12]. In the early 1980s, Sundaresan et al*.* and White et al*.* presented retrospective clinical studies regarding the surgical resection of BMs [13, 14], while two randomized trials in the early 1990s demonstrated that surgical resection was associated with better outcomes in patients with BMs [6, 15]. In addition, surgical resection for BMs has developed significantly during the last three decades, with advancements in new techniques and technologies.[16] We considered that the indications and contraindications for surgical resection thus need to be revisited in light of the evolution of the procedure and its improved effectiveness for BMs.

Surgical resection of BMs is traditionally carried out in patients with good KPS [16–18]; however, there is little direct evidence regarding the suitability of surgical resection in patients with BMs and poor KPS. We conducted a multicenter, retrospective cohort study to determine whether neurosurgical resection provided a survival benefit in patients with lung cancer BMs and poor KPS.

## Materials and methods

### Study population

This multicenter, retrospective cohort study included 386 patients with lung cancer BMs with pretreatment KPS ≤ 70, among a total of 1177 patients with lung cancer BMs. All patients were treated at two tertiary cancer centers and one tertiary comprehensive hospital in southern China between August 10, 2010 and July 1, 2021. The study was approved by the institutional review board of Sun Yat-sen University Cancer Center, which waived the need for informed consent. This study has been reported in line with the STROCSS criteria [19] and was approved by the Medical Ethics Committees of SYSUCC (Reference No. B2020- 218–01). In addition, this study was registered at ClinicalTrials.gov (identifier: NCT05609162. https://clinicaltrials.gov/).

### Patient selection

The inclusion criteria were: (1) pathological evidence of primary lung cancer; (2) BMs confirmed by enhanced MRI; and (3) availability of complete clinical information. The exclusion criteria were: (1) patients with two or more types of cancer; (2) overall survival (OS) < 1 month; (3) patients receiving ventricle puncture surgery or other non-surgical resection; and (4) multiple encounters for the same patient. Data for the initial visit were used in the analysis for patients with multiple visits. Furthermore, we considered that 1 month was too short as an exposure period, and we therefore excluded patients with an OS time of < 1 month. Among all the patients with lung cancer BMs, patients with pretreatment KPS ≤ 70 were finally selected and divided into a surgical resection group (surgery group) and a non-surgical resection group (non-surgery group). We defined a pretreatment KPS ≤ 70 as a poor KPS because such a score was associated with poor outcomes in patients with lung cancer BMs in a previous study [10].

### Outcomes

The main outcome was OS after diagnosis of BM. OS was defined as the time from the date of BM diagnosis to the date of last follow-up or date of death or censoring. Follow-up data were collected at clinical visits and by telephone consultations.

### Covariates

Baseline characteristics including age, sex, smoking history, histology, epidermal growth factor receptor (*EGFR*)/anaplastic lymphoma kinase (*ALK*) status, extracranial metastases, synchronous metastases, location of BMs, number of BMs, radiotherapy (Whole brain radiotherapy, WBRT; stereotactic radiosurgery SRS; WBRT + SRS), chemotherapy and target therapy or immunotherapy were collected from the hospital information system and medical records.

### Statistical analysis

We adjusted the differences of covariates and eliminated the potential bias between the surgery and non-surgery groups using two propensity score (PS)-based adjustment methods: propensity score matching (PSM) and inverse probability of treatment weighting (IPTW) [20, 21]. The PS for each participant was calculated from logistic regressions including baseline covariates. We applied the inverse PS as a weight for the surgery group, and the inverse of 1 − PS for the non-surgery group. In addition, the PS of PSM was calculated with a ratio of 1:1 and a caliper width of 0.02. *P* value ≥ 0.05 was used to assess the balance of between-group differences after IPTW or PSM adjustment.

Clinical characteristics were compared between the surgery and non-surgery groups using χ^2^ tests for categorical variables. Differences in OS were compared using Kaplan–Meier analysis and the log-rank test. Risk factors for OS were evaluated by univariate and multivariate Cox proportional hazards regression analyses. All characteristics were included in a multivariate Cox regression model. Analyses were performed using SPSS version 26.0 (IBM, Armonk, NY, USA) and R studio (version 1.1.383) with R CRAN (v.4.2.1, R Core Team 2022). All statistical tests were two-sided, and *P* < 0.05 was considered statistically significant.

#### Sensitivity analyses

We conducted sensitivity analyses to examine the robustness of our results. We first used a multivariate Cox proportional hazards regression model to assess the hazard ratios (HRs) with 95% confidence intervals (CIs) for the risk factors of OS in the unadjusted data. We then carried out IPTW to adjust the differences in covariates between the surgery and the non-surgery groups. We also used the 1:1 PSM method to mimic the conditions of randomized clinical trials and reduce selection bias between the two groups [22, 23]. Finally, we used a multivariate Cox proportional hazards regression model after IPTW or PSM adjustment to further verify the results.

## Results

**Fig. 1:**
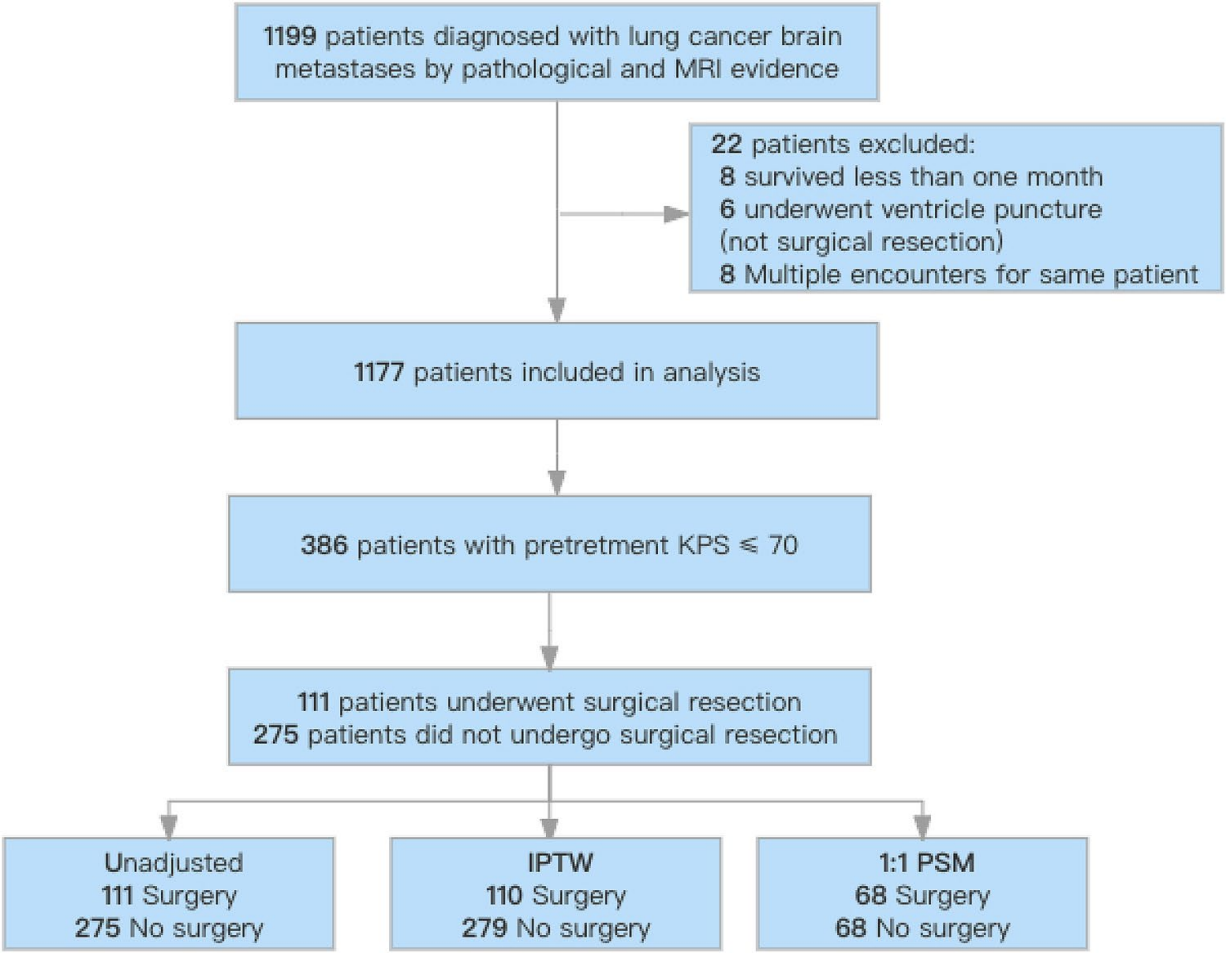
Study flow diagram. IPTW, inverse probability of treatment weighting; PSM, propensity score matching

**Fig. 2:**
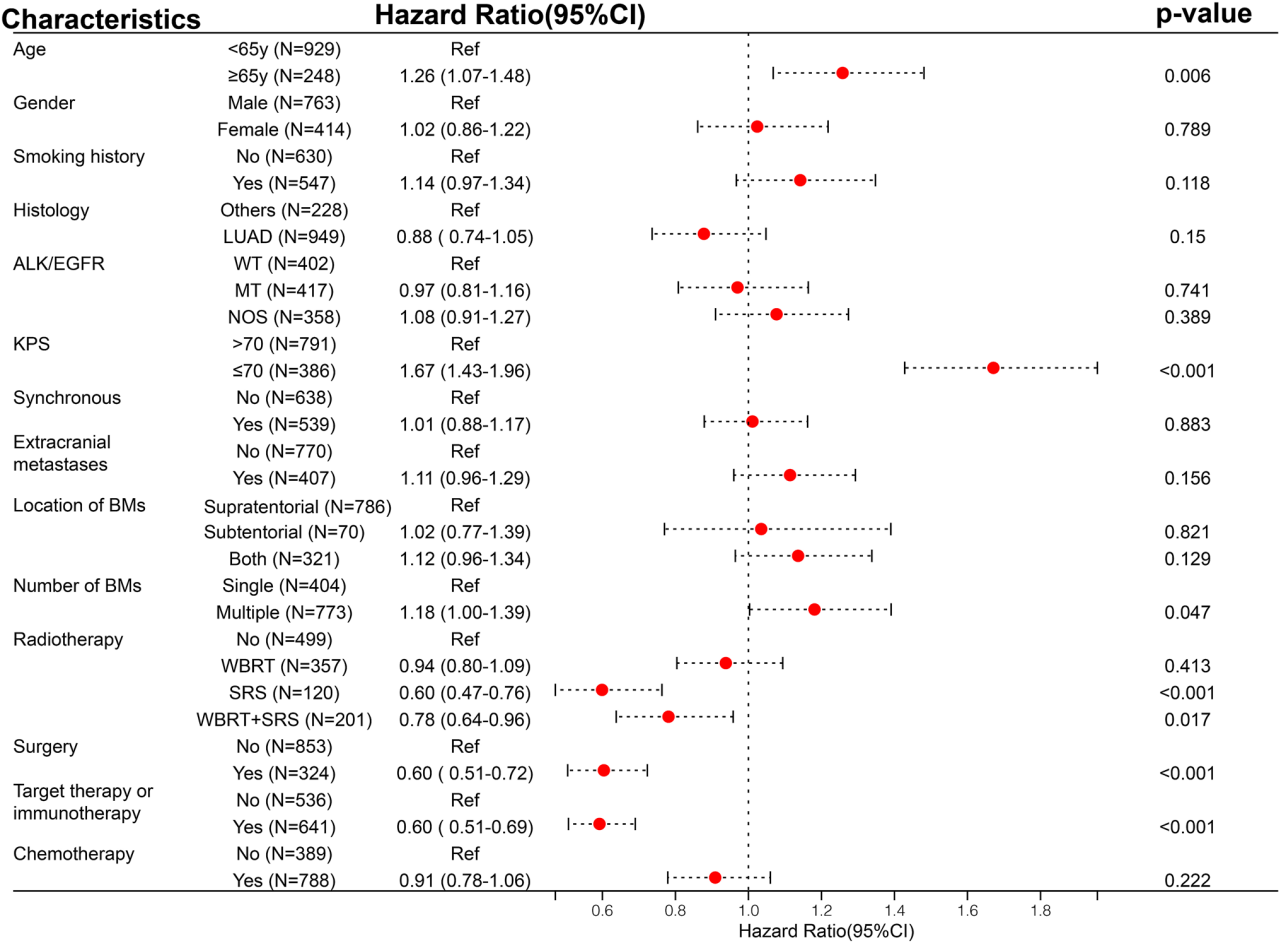
Forest map of multivariate COX regression analysis in 1177 lung cancer brain metastases

Study flow diagram was shown in Fig. 1. Multivariate Cox regression analysis of all 1177 patients with BMs indicated that a pretreatment KPS ≤ 70 was related to poor outcome (HR: 1.71, 95%CI: 1.47–2.00, *P* < 0.001; Fig. 2). We therefore further analyzed 386 patients with a pretreatment KPS ≤ 70 (mean age [SD], 57.85 [10.36] years; mean KPS [SD], 60.91 [10.11]), including 111 patients who underwent neurosurgical resection and 275 patients who did not. The baseline characteristics including age, smoking history, synchronous metastases, location of BMs, number of BMs, and radiotherapy, chemotherapy and target therapy or immunotherapy differed significantly between the two groups in the unadjusted cohort. The percentages of patients aged ≥ 65 years, smoking history, radiotherapy, chemotherapy and target therapy or immunotherapy were lower in the surgery group compared with the non-surgery group, whereas the percentages of synchronous metastases, supratentorial BMs, and single BMs were higher in the surgery group compared with the non-surgery group (Table 1). However, the baseline characteristics were balanced between the two groups after adjustment with IPTW or PSM (Table 1).

**Table 1:** Characteristics of lung cancer BMs with KPS ≤ 70 stratified by neurosurgical resection

Characteristics	Unadjusted No. (%)			PSM^a^_ajusted No. (%)			IPTW^j^_ajusted No. (%)		
	No (n = 275)	Surgery (n = 111)	*P*-value	No (n = 68)	Surgery (n = 68)	*P*-value	No (n = 279)	surgery (n = 110)	*P*-value
Age			0.02			0.67			0.1
< 65y	197 (71.6)	93 (83.8)		53 (77.9)	56 (82.4)		212 (76.0)	94 (85.5)	
≥ 65y	78 (28.4)	18 (16.2)		15 (22.1)	12 (17.6)		67 (24.0)	16 (14.5)	
Gender			0.49			1			0.34
Male	195 (70.9)	74 (66.7)		45 (66.2)	46 (67.6)		192 (67.7)	66 (60.0)	
Female	80 (29.1)	37 (33.3)		23 (33.8)	22 (32.4)		87 (32.3)	34 (40.0)	
Smoking history			0.01			1			0.92
No	114 (41.5)	62 (55.9)		37 (54.4)	38 (55.9)		131 (47.0)	50 (45.5)	
Yes	161 (58.5)	49 (44.1)		31 (45.6)	30 (44.1)		148 (53.0)	60 (54.5)	
Histology			0.86			0.46			0.66
LUAD^d^	199 (72.4)	82 (73.9)		49 (72.1)	44 (64.7)		198 (71.0)	74 (67.3)	
Others	76 (27.6)	29 (26.1)		19 (27.9)	24 (35.3)		81 (29.0)	36 (32.7)	
EGFR/ALK			0.19			0.67			0.76
WT^e^	99 (36.0)	31 (27.9)		24 (35.3)	21 (30.9)		98 (35.1)	45 (40.9)	
MT^f^	75 (27.3)	29 (26.1)		22 (32.4)	20 (29.4)		71 (25.4)	27 (24.5)	
NOS^g^	101 (36.7)	51 (45.9)		22 (32.4)	27 (39.7)		110 (39.5)	38 (44.6)	
Synchronous			0.004			0.72			0.96
No	119 (43.3)	30 (27.0)		21 (30.9)	24 (35.3)		109 (39.1)	42 (38.2)	
Yes	156 (56.7)	81 (73.0)		47 (69.1)	44 (64.7)		170(60.9)	68 (61.8)	
Extracranial metastases			0.21			0.16			0.91
No	227 (82.5)	98 (88.3)		64 (94.1)	58 (85.3)		236 (84.6)	92 (83.6)	
Yes	48 (17.5)	13 (11.7)		4 (5.9)	10 (14.7)		43 (15.4)	18 (16.4)	
Location of BMs^h^			< 0.001			0.52			0.95
Supratentorial	135 (49.1)	73 (65.8)		44 (64.7)	42 (61.8)		152 (54.5)	61 (63.1)	
Subtentorial	16 (5.8)	12 (10.8)		5 (7.4)	9 (13.2)		23 (8.2)	10 (13.8)	
Both	124 (45.1)	26 (23.4)		19 (27.7)	17 (25)		104 (37.3)	29 (23.1)	
Number of BMs			< 0.001			0.58			0.66
Single	62 (22.5)	46 (41.4)		20 (29.4)	24 (35.3)		78 (28.0)	28 (25.5)	
Multiple^i^	213 (77.5)	65 (58.6)		48 (70.6)	44 (64.7)		201 (72.0)	82 (74.5)	
Radiotherapy			< 0.001			0.56			0.58
No	44 (16.0)	73 (65.8)		26 (38.2)	34 (50)		88 (31.5)	33 (30)	
WBRT	90 (32.7)	27 (24.3)		30 (44.1)	23 (33.8)		82 (29.4)	29 (26.4)	
SRS	35 (12.7)	4 (3.6)		4 (5.9)	4 (5.9)		28 (10.0)	15 (13.6)	
WBRT + SRS	106 (38.5)	7 (6.3)		8 (11.8)	7 (10.3)		81 (29.1)	33 (30)	
Chemotherapy			0.004			0.73			0.68
No	106 (38.5)	61 (55)		35 (51.5)	32 (47.1)		122 (43.7)	45 (40.9)	
Yes	169 (61.5)	50 (45)		33 (48.5)	36 (52.9)		157 (56.3)	65 (59.1)	
Target therapy or immunotherapy			< 0.001			0.49			0.87
No	111 (40.4)	70 (63.1)		40 (58.8)	35 (51.5)		136 (48.7)	55 (50.0)	
Yes	164 (59.6)	41 (36.9)		28 (41.2)	33 (48.5)		143 (51.3)	55 (50.0)	

^a^Propensity score matching; ^b^whole brain radiotherapy; ^c^stereotactic radiosurgery; ^d^lung adenocarcinoma; ^e^wild type; ^f^mutation; ^g^unknown or untested; ^h^brain metastases, ^i^number of BMs ≥ 2; ^j^ Inverse probability treatment weighting

All characteristics were included in the multivariate Cox regression model (Table 2). In the unadjusted cohort, SRS, surgery and target therapy or immunotherapy were associated with a significantly better prognosis (SRS, HR: 0.56, 95%CI: 0.34–0.91, *P* = 0.02; surgery, HR: 0.68, 95%CI: 0.51–0.91, *P* = 0.01; target therapy or immunotherapy, HR: 0.70, 95%CI: 0.53–0.93, *P* = 0.01, Tables 2). After PSM, only surgery was associated with a significantly better prognosis (HR: 0.61, 95%CI: 0.39–0.94, *P* = 0.03, Table 2). After IPTW, chemotherapy and surgery were associated with significantly better prognoses (chemotherapy, HR: 0.70, 95%CI: 0.52–0.95, *P* = 0.02; surgery, HR: 0.58, 95%CI: 0.40–0.84, *P* = 0.004, Table 2).

**Table2:** Multivariate analyses of risk factors for OS lung cancer BMs with KPS ≤ 70

Covariates	Level	Unadjusted		After PSM		After IPTW	
		HR (95% CI)	*P*-value	HR (95% CI)	*P*-value	HR (95% CI)	*P*-value
Age	< 65y	Reference		Reference		Reference	
	≥ 65y	1.26 (0.96–1.64)	0.10	1.27 (0.77–2.10)	0.35	1.36 (0.97–1.90)	0.07
Gender	Male	Reference		Reference		Reference	
	Female	0.98 (0.72–1.34)	0.90	1.0 (0.61–1.62)	1.0	1.02 ( 0.65–1.60)	0.95
Smoking history	No	Reference		Reference		Reference	
	Yes	1.27 (0.95–1.70)	0.11	1.24 (0.78–1.96)	0.36	1.29 ( 0.87- 1.92)	0.21
Histology	LUAD	Reference		Reference		Reference	
	Others	0.91 (0.70–1.20)	0.51	0.93 (0.58–1.51)	0.78	0.87 (0.60–1.25)	0.44
EGFR/ALK	WT	Reference		Reference		Reference	
	MT	1.08 (0.78–1.50)	0.65	1.57 (0.87–2.85)	0.14	1.15 (0.75–1.76)	0.53
	NOS	1.05 (0.79–1.40)	0.76	1.08 (0.64–1.82)	0.78	1.34 (0.88- 2.03)	0.17
Synchronous	No	Reference		Reference		Reference	
	Yes	0.96 (0.76–1.22)	0.73	1.09 (0.70–1.70)	0.67	0.89 (0.66–1.19)	0.44
Extracranial metastases	No	Reference		Reference		Reference	
	Yes	0.94 (0.68–1.30)	0.71	0.79 (0.37–1.71)	0.55	0.90 (0.61–1.32)	0.58
Location of BMs	Supratentorial	Reference		Reference		Reference	
	Subtentorial	0.78 (0.47–1.28)	0.33	1.13 (0.54–2.35)	0.74	0.72 (0.38–1.34)	0.3
	Both	1.19 (0.91–1.56)	0.21	1.21 (0.74–1.99)	0.45	1.34 (0.96–1.88)	0.09
Number of BMs	Single	Reference		Reference		Reference	
	Multiple	1.00 (0.74–1.36)	0.99	0.99 (0.60–1.62)	0.96	0.84 (0.56–1.26)	0.39
Radiotherapy	No	Reference		Reference		Reference	
	WBRT	1.19 (0.86–1.65)	0.28	1.05 (0.64–1.73)	0.84	1.27 (0.88–1.83)	0.2
	SRS	0.56 (0.34–0.91)	0.02	0.57 (0.21–1.56)	0.27	0.52 (0.25–1.09)	0.08
	WBRT + SRS	1.01 (0.70–1.45)	0.96	1.02 (0.51–2.02)	0.97	1.05 (0.68–1.60)	0.84
Surgery	No	Reference		Reference		Reference	
	Yes	0.68 (0.51–0.91)	0.01	0.61 (0.39–0.94)	0.03	0.58 (0.40–0.84)	0.004
Chemotherapy	No	Reference		Reference		Reference	
	Yes	0.79 (0.61–1.03)	0.08	0.67 (0.43–1.02)	0.06	0.70 (0.52–0.95)	0.02
Target therapy or immunotherapy	No	Reference		Reference		Reference	
	Yes	0.70 (0.53–0.93)	0.02	0.63 (0.37–1.08)	0.09	0.85 (0.59–1.24)	0.4

**Fig. 3:**
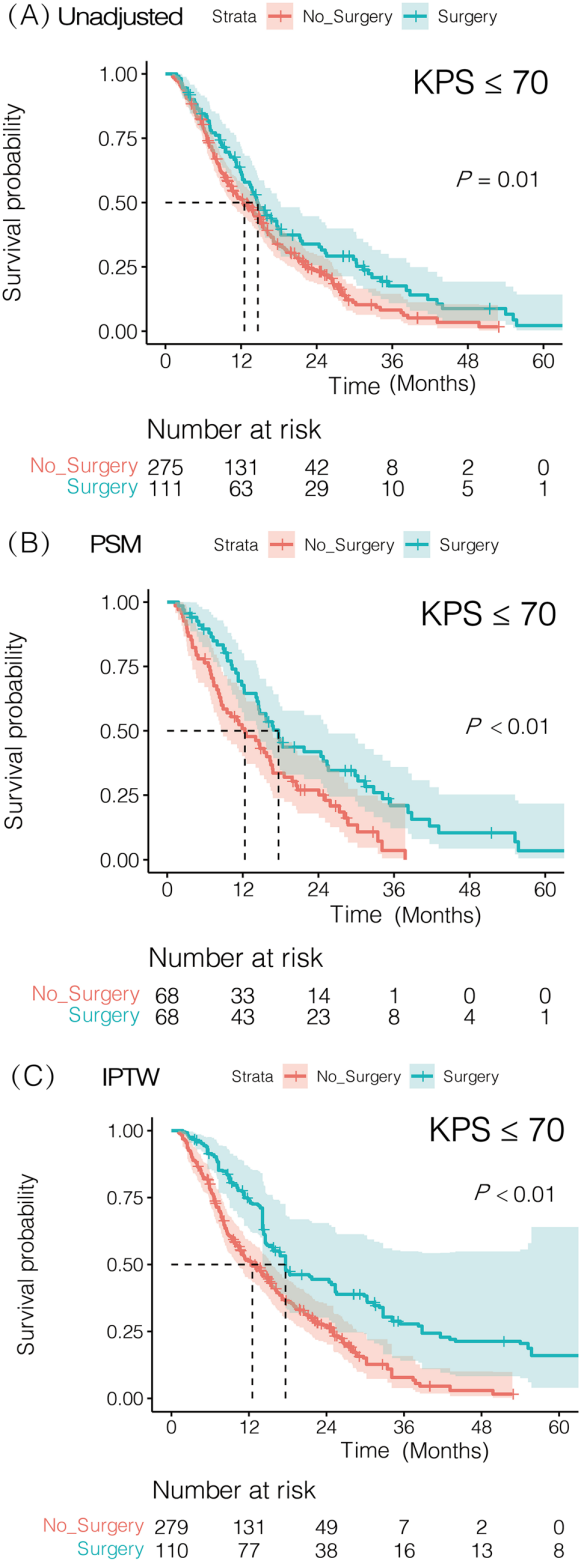
Kaplan–Meier overall survival (OS) curves of lung cancer BM with KPS ≤ 70 stratified by surgical resection. **A** Kaplan–Meier OS curves unadjusted. **B** Kaplan–Meier OS curves after PSM. **C** Kaplan–Meier OS curves after IPTW

In the Kaplan–Meier survival plot (Fig. 3), patients in the surgery group had significantly longer OS than patients in the non-surgery group, using unadjusted data (median OS, 14.7 vs 12.5 months, *P* = 0.01), PSM-adjusted data (median OS, 17.7 vs 12.3 months, *P* < 0.01), and IPTW-adjusted data (median OS, 17.7 vs 12.5 months, *P* < 0.01). In lung adenocarcinoma or squamous carcinoma BMs with KPS ≤ 70, neurosurgical resection was associated with a good prognosis after PSM or IPTW adjustment (Supplementary, Figure S1, Figure S2).

## Discussion

The results of this multicenter, retrospective cohort study showed that neurosurgical resection improved OS and was associated with a significantly better prognosis in patients with lung cancer BMs and poor KPS. Differences in baseline characteristics between the two groups were balanced and the association between neurosurgical resection and improved OS remained evident after IPTW or PSM adjustment.

As most of BMs are located in the cerebral cortex, up to 40% of BMs present with focal neurological deficits, while increased intracranial pressure from mass effect and vasogenic edema is also common. This often leads to altered mental status or impaired cognition, which makes KPS worse [1]. Neurosurgical resection can rapidly relieve symptoms of intracranial hypertension, and reduce focal neurological deficits [16]. Besides, the lesions of BMs are usually well circumscribed and wrapped by gliotic pseudo capsule, which is contrary to the diffuse and invasive characteristics of primary brain tumors [16]. Thus, gross total resection (GTR) was reported to improve the outcome of BMs [24]. Various technologies can assist the neurosurgeon to achieve gross total resection, such as preoperative functional MRI, intraoperative neuronavigation, cortical brain mapping, intraoperative ultrasound, and fluorescence-guidance [25–29]. Therefore, these evidence may support our findings that surgical resection provides survival benefit in the setting of poor KPS. However, there is still no direct evidence published on that whether surgical resection is appropriate for BMs with poor KPS.

European Association of Neuro-Oncology guidelines recommend that surgical resection can be performed in patients with BMs and a KPS ≥ 60 and controlled systemic disease [18], while the National Comprehensive Cancer Network Guidelines for Central Nervous System Cancers recommend radiotherapy rather than surgical resection for BMs in patients with systemic disease progression and a poor KPS [30]. These recommendations were based on the results of a phase III randomized controlled trial of 84 patients with BMs, active systemic disease, and low KPS (mean KPS 77.63). Surgery plus WBRT showed no OS benefit compared with WBRT alone, suggesting that surgery plus WBRT might be limited to patients with stable systemic disease and a good KPS [31]. However, in contrast to this previous trial [31] and the guidelines [18, 30], the current results indicated that surgical resection improved OS. This apparent discrepancy may be attributable to several factors. First, our results were based on multicenter data involving more participants than the previous randomized controlled trial. Second, we only selected patients with lung cancer BMs, while the trial included patients with BMs from a variety of primary cancers. Third, we divided patients into surgery and non-surgery groups, while the patients in the previous trial were divided into surgery plus WBRT and WBRT alone groups. Fourth, the mean KPS of all patients in our study was 60.91 (range: 30–70), compared with 77.63 (range: 50–100) in the trial. Fifth, our study was non-randomized, although we used PSM adjustment to mimic a randomized controlled trial. Sixth, the covariates differed between the two studies. Finally, the trial was conducted in the 1990s, and surgical techniques have since evolved significantly during the 2020s [31].

Despite the positive effect of surgical resection on OS in patients with lung cancer BM and a poor KPS in the current study, the response to surgical resection may differ among individuals, and surgical candidates should thus be selected carefully. Potential prognosis factors should be considered, including age, number of BMs, neurocognitive function, status of the primary cancer and systemic disease, genetic testing, and routine preoperative examination [16].

This study had several strengths. First, to the best of our knowledge, this was the first multicenter, retrospective study to investigate the survival benefit of neurosurgical resection in patients with lung cancer BMs and poor KPS, thus providing a clinical reference. Second, we performed IPTW and PSM to adjust for various potentially confounding factors, and showed that the baseline characteristics were balanced after adjustment. Third, we performed multivariate Cox regression models before and after IPTW and PSM adjustment, to verify the results. All the results consistently indicated that neurosurgical resection was an independent protective factor for OS, suggesting that the study results were robust.

However, the study also had some limitations. First, it was a non-randomized study and potential flaws might have remained, despite IPTW and PSM. Second, after PSM, nearly half of the patients were unmatchable and excluded, probably due to differences in covariates between the two groups.

## Conclusions

Neurosurgical resection improved OS and was associated with a significantly better prognosis in patients with lung cancer BMs and poor KPS. These findings suggest that a poor KPS should not be a contraindication for surgical resection in patients with lung cancer BMs.

## Supplementary Information

Below is the link to the electronic supplementary material.

Supplementary file1 (DOCX 2437 KB)

## Data Availability

As the study involved the privacy of a large population, raw data would remain confidential and would not be shared. If you need relevant data, please contact the corresponding author through email.
