# Supplementary material for: Poor Karnofsky performance status is not a contraindication for neurosurgical resection in patients with lung cancer brain metastases: a multicenter, retrospective PSM-IPTW cohort study
Source: J Neurooncol. 2023 Mar 20;162(2):327–35. doi: 10.1007/s11060-023-04293-8 (PMC10167153; doi:10.1007/s11060-023-04293-8)
Supplement: Supplementary file 1 — Supplementary file1 (DOCX 2437 KB) [file 11060_2023_4293_MOESM1_ESM.docx]

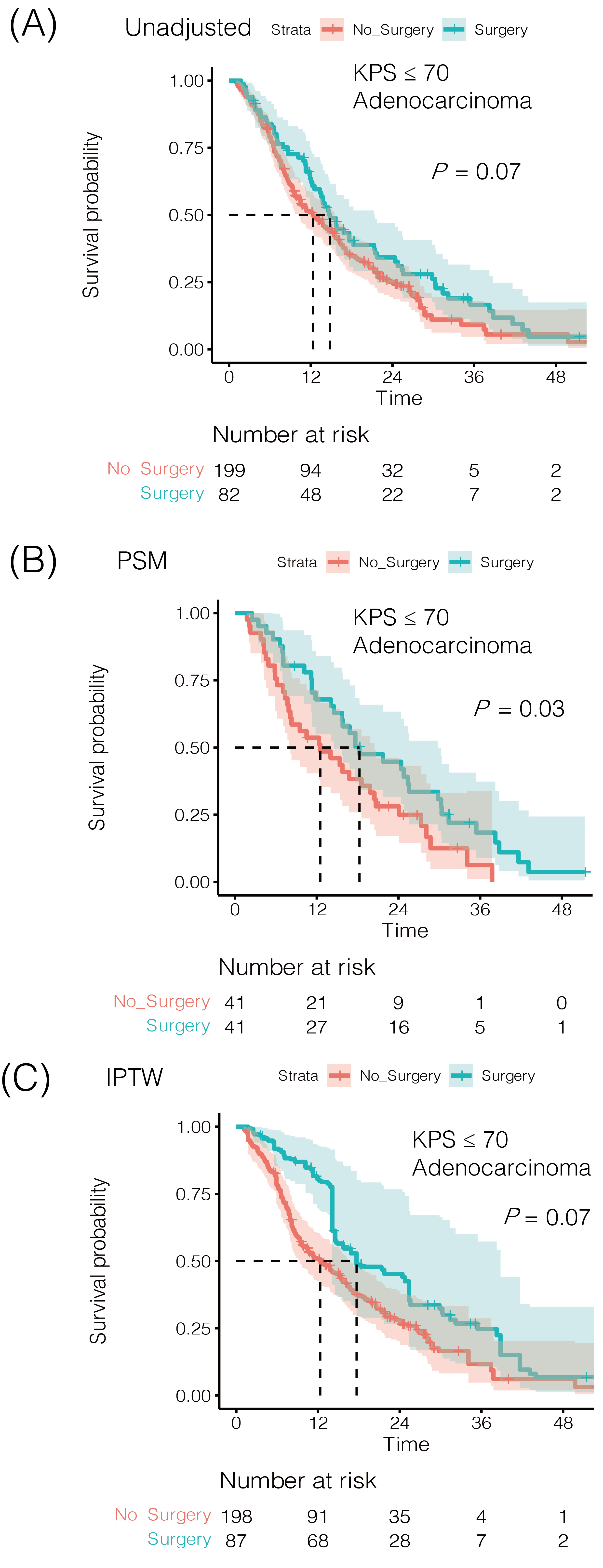


Figure S1. Kaplan–Meier overall survival (OS) curves of lung adenocarcinoma BM with KPS ≤ 70 stratified by neurosurgical resection. (A) Kaplan–Meier OS curves unadjusted. (B) Kaplan–Meier OS curves after PSM. (C) Kaplan–Meier OS curves after IPTW.


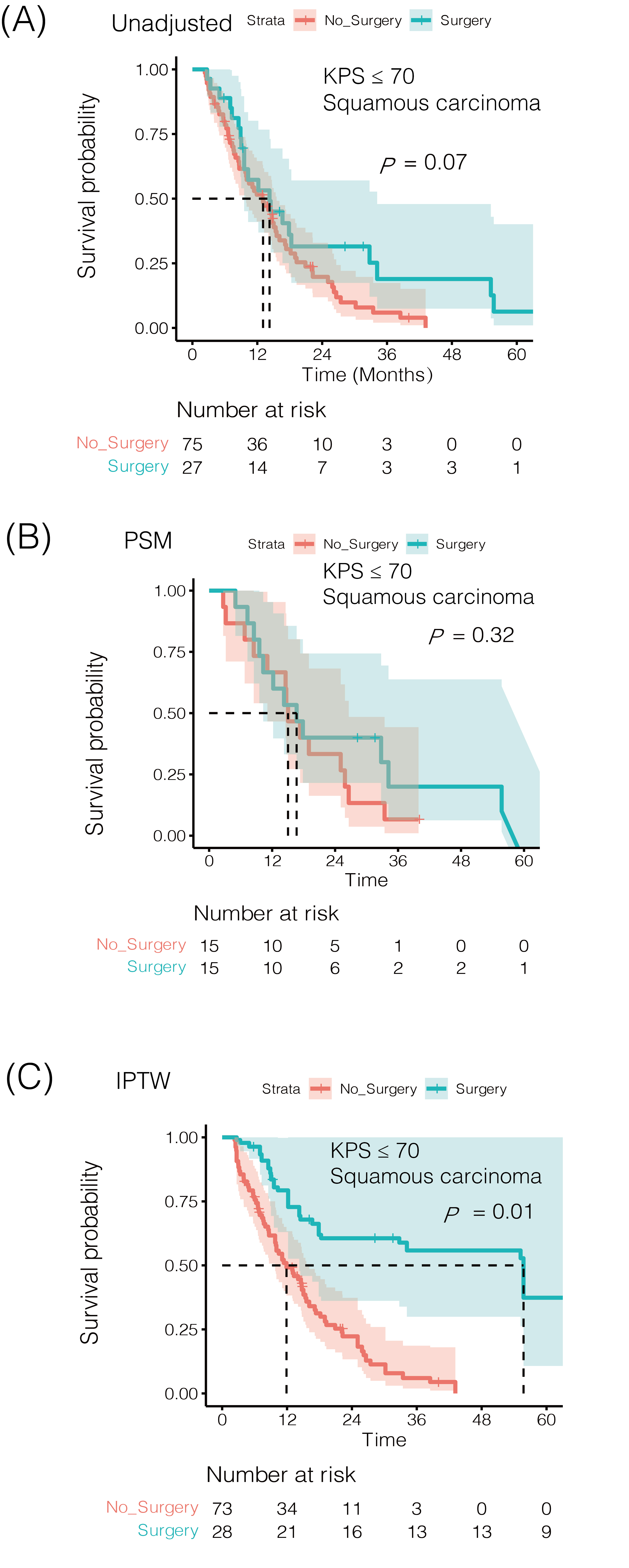


Figure S2. Kaplan–Meier overall survival (OS) curves of lung squamous carcinoma BM with KPS ≤ 70 stratified by neurosurgical resection. (A) Kaplan–Meier OS curves unadjusted. (B) Kaplan–Meier OS curves after PSM. (C) Kaplan–Meier OS curves after IPTW.
